# Dry eye disease and depression-anxiety-stress: A hospital-based case control study in Turkey

**DOI:** 10.12669/pjms.313.7091

**Published:** 2015

**Authors:** Ugur Yilmaz, Mehmet Enes Gokler, Alaettin Unsal

**Affiliations:** 1 Ugur Yilmaz, MD, Specialist Ophthalmologist, Nigde State Hospital Eye Clinic, Nigde, Turkey; 2 Mehmet Enes Gökler, MD, Research Assistant, Department of Public Health, Eskisehir Osmangazi University, Medical Faculty, Eskisehir, Turkey; 3 Alaettin Unsal, MD, Professor, Department of Public Health, Eskisehir Osmangazi University, Medical Faculty, Eskisehir, Turkey

**Keywords:** Dry eye disease, Depression, Anxiety, Stress, Turkey

## Abstract

**Objective:**

The aim of the present study was to investigate the association between dry eye disease (DED) and psychosomatic conditions, such as depression, stress, and anxiety, and the distribution of associated risk factors.

**Methods:**

In this case control study, the sample consisted of 121 DED subjects and 242 control subjects. Each subjects was diagnosed as having DED or not by an ophthalmologist. Ocular Surface Disease Index and Depression Anxiety Stress Scales were administered to all subjects. Data were analysed using chi-square and Mann Whitney U tests as a univariate analysis and multiple logistic regression as a multivariate analysis.

**Results:**

Of 1,458 consecutive outpatients, clinically diagnosed DED was present in 121 individuals (8.3%). There was a significant relationship of family history of DED (OR, 1.43; 95% CI, 0.84-2.41), chronic disease history (OR, 2.84; 95% CI, 1.66-4.87), OSDI score (OR, 1.07; 95% CI, 1.97–4.06), depression (OR, 2.06; 95% CI, 1.30-3.27), anxiety (OR, 2.66; 95% CI, 1.67-4.23), and stress (OR, 2.33; 95% CI, 1.48-3.67) with DED.

**Conclusion:**

Individuals with depression, anxiety and stress are more likely to experience DED. In addition to confirming some well-known risk factors, this study has found new associations between DED and a family history of DED and the presence of stress.

## INTRODUCTION

Dry eye disease (DED) is a multifactorial disease that affects tears and the ocular surface, and it results in damage to the ocular surface according to the International Dry Eye Work Shop in 2007.^1^ DED is an important public health problem and causes ocular discomfort and visual disturbances that may interfere with daily activities such as reading, working on a computer and driving.^2^ In addition, the symptoms of DED can progress to complications such as corneal epithelial defects, recurrent conjunctivitis, corneal ulceration, corneal scarification or perforation, and loss of vision.^2^

Globally, the prevalence of DED varies within a wide range depending on the definition of DED, age distribution of the population and the methodology. The prevalence of dry eye disease ranges from 5.5% to 50.1% in community-based epidemiological studies^3^-^6^ and ranges from 7.99% to 29.9% in some hospital-based studies.^7^-^11^

The prevalence of DED may vary with sex, age and geography. The disease prevalence seems to be higher in females, elderly individuals and Asians.^1^,^5^,^6^ It is important to understand the impact of factors that could affect the ocular surface and cause dry eye symptoms. Studies have shown that hormonal changes, smoking, ocular surgery, medications, indoor pollutants, low humidity, high room temperature and contact lens wear are risk factors of DED.^1^,^11^

The symptoms of dry eye disease include burning, itching, redness, the sensation of a foreign body sensation object in the eye, and pain. These symptoms are rarely severe but decrease the individual’s quality of life and have a negative impact on the individual’s mood and mental health.^2^,^12^ Studies have shown that the symptom severity is not only associated with corneal sensations or ocular surface damage, but is also associated with individual pain perception or psychosomatic conditions such as depression, stress, and anxiety.^13^,^14^ Additionally, patients with depression or anxiety may have a greater likelihood of developing DED due to antianxiety and antidepressant medications.^15^

The aim of the present study was to investigate the association between DED and psychosomatic conditions, such as depression, stress, and anxiety, and the distribution of associated risk factors among a hospital-based population in Turkey.

## METHODS

The study consisted of a hospital-based case control study conducted at the secondary care hospital in Nigde, Turkey. All consecutive individuals with dry eye symptoms (age ≥18 years) who were examined and diagnosed DED in the Eye clinic between March 1 and September 30, 2014 were enrolled, the case group. The control group, consisted of individuals admitted to other clinics in the same hospital. Individuals in the control group were excluded from study if they had experienced any eye problems in the last 6 months. As a result, while patients in the case group had received a diagnosis of dry eye, there were no dry eye patients in the control group. The study design was approved by the Eskisehir Osmangazi University Ethics Committee, and all individuals provided written informant consent.

The goal of our study was to include 121 cases. In this study, the ratio of controls to cases was 2. To ensure similarity between the case group and the control group, cases were ‘matched’ to controls in terms of age and sex.

DED was diagnosed according to the presence of dry eye symptoms, the tear film breakup time test (TBUT) and the Schirmer test (ST). An ST result of <5 mm and/or a TBUT result of <10 s were considered pathological. All tests were performed by a specialized ophthalmologist. The Ocular Surface Disease Index (OSDI) questionnaire was used for DES symptoms. The OSDI was developed in 1997 by Walt et al., and the reliability and validity studies for the Turkish version of the OSDI were performed in 2007 by Ozcura et al.^16^,^17^ This questionnaire included questions regarding the frequency of dry eye symptoms experienced in the previous week (light sensitivity, gritty sensation, painful or sore eyes, blurred vision, and poor vision), vision related daily activities (reading, watching TV, working on computers, and driving at night) and environmental triggers (wind, air conditioning, and low humidity). Each answer was scored on a 5-point scale (all of the time-4, most of the time-3, half of the time-2, some of time-1 and none of the time-0), and the OSDI score was calculated as follows: {(sum of scores×25) /total number of questions}. Thus the total OSDI score ranged from 0 to 100. A higher OSDI score represented greater disability.

To measure anxiety, depression and stress, we used the Depression Anxiety Stress Scales (DASS). The DASS was developed in 1995 by Lovibond et al., and the reliability and validity studies for the Turkish version of the DASS were performed by Akin and Çetin in 2007.^18^,^19^ The DASS contains 42 questions, 14 for each of the three subscales consisting of depression, anxiety and stress. Individuals indicate the extent to which they have experienced each of the symptoms depicted in the items during the previous week on a 4-point Likert-type scale ranging from 0 (did not apply to me at all) to 3 (applied to me very much or most of the time). For each scale, the total score ranged from 0 to 42. Scores that were considered in the normal range included 0-9 for depression, 0-7 for anxiety, and 0-14 for stress. If the total score was higher than these limits, it was considered suggestive of depression, anxiety or stress.

The questionnaire consisted of two parts. The first part of the questionnaire included the individuals’ socio-demographic characteristics (age, gender, education level, employment status, income level, cigarette and alcohol habits, chronic disease history, height and weight) and some factors thought to be associated with dry eye (visual display terminal use, contact lens/glasses use, and family history of DED). The second part of the questionnaire included the OSDI and DASS questions, which were used to determine the presence of depression, anxiety and stress.

We categorised BMI in to 3 groups according to the WHO standards (<18.5, 18.5-24.9, and ≥25.0) and defined visual display terminal (VDT) use (none, <2, 2-4, and>4 hours), alcohol drinking (current drinker or not), smoking (current smoker or not) and income level (bad, fair and good, according to the patient’s perception).

The statistical analyses were conducted with IBM SPSS Statistics software (version 20.0). Data were analysed using chi-square and Mann Whitney U tests as a univariate analysis and multiple logistic regression as a multivariate analysis. First, we conducted the univariate analysis. As a second step, binary logistic regression adjusted for age, sex and education was used to examine the associations between DED and other potential risk factors (variables at a p<0.20 significance level in the univariate analysis). A value of p<0.05 was considered statistically significant.

## RESULTS

Of 1,458 consecutive outpatients, clinically diagnosed DED was present in 121 individuals (8.3%). In this study, 363 participants were included, 121 in the case group and 241 in the control group. Of these, 75.5% of the participants were female in the case group and 74.4% were female in the control group (p>0.05). The mean age of the case group was 42.3 ± 12.7 years, and the mean age of the control group was 42.4 ± 12.5 years (p>0.05). Characteristics of the study population in the case and control groups are shown in Table-I.

**Table-I:** Characteristics of the Study Population in the case and control groups.

			Case Group (N: 121)	Control Group (N: 242)	Test Z/X^2^;p
Age (years)		Median (SD)	41.0	40.0	0.164; 0.870
		IQR 25-75	33-52	34-52	
Sex					
	Male	n (%)	27 (22.3)	62 (25.6)	0.476;0.490
	Female	n (%)	94 (77.7)	180 (74.4)	
Education					
	No education or primary school	n (%)	52 (43.0)	102 (42.1)	0.895; 0.639
	Secondary school	n (%)	15 (12.4)	23 (9.5)	
	High school or University	n (%)	54 (44.6)	117 (48.3)	
Income level					
	Bad	n (%)	11 (9.1)	38 (15.7)	4.048; 0.132
	Fair	n (%)	86 (71.1)	149 (61.6)	
	Good	n (%)	24 (19.8)	55 (22.7)	
Current Smoker					
	No	n (%)	95 (78.5)	174 (71.9)	1.838; 0.175
	Yes	n (%)	26 (21.5)	68 (28.1)	
Current alcohol drinker					
	No	n (%)	119 (98.3)	235 (97.1)	0.513; 0.474
	Yes	n (%)	2 (1.7)	7 (2.9)	
Family history of DED					
	No	n (%)	94 (77.7)	224 (92.6)	16.438; <0.001
	Yes	n (%)	27 (22.3)	18 (7.4)	
Chronic disease history					
	No	n (%)	60 (49.6)	165 (68.2)	11.837; 0.001
	Yes	n (%)	61 (50.4)	77 (31.8)	
Body mass index (kg/m^2^)					
	<18.5	n (%)	3 (2.5)	4 (1.7)	0.426; 0.808
	18.5-24.9	n (%)	49 (40.5)	94 (38.8)	
	≥25.0	n (%)	69 (57.0)	144 (59.5)	
VDT use (hours/day)					
	No	n (%)	1 (0.8)	9 (3.7)	7.669; 0.053
	0-2	n (%)	11 (9.1)	42 (17.4)	
	2-4	n (%)	50 (41.3)	93 (38.4)	
	≥4	n (%)	59 (48.8)	98 (40.5)	
OSDI score		Median (SD)	20 (8.5)	10 (8.3)	9.063: <0.001
		IQR 25-75	15.5-28	5-17	

IQR: interquartile range

Defined depression, anxiety and stress were more prevalent in patients diagnosed with DED than in subjects without DED. The mean scores of the case group with regard to the depression, anxiety and stress subscales were 10.96±7.25 (range 0-39), 11.41±7.33 (range 0-39), and 14.50±7.44 (range 0-40) points, respectively. The mean scores of the control group with regard to the depression, anxiety and stress subscales were 8.18±7.12 (range 0-30), 7.20±6.50 (range 0-28), and 11.08±7.69 (range 0-33) points, respectively. The distributions of depression, anxiety and stress in the case and control groups are shown in Table-II.

**Table-II:** The distributions of depression, anxiety and stress in the case and control groups.

			Case Group	Control Group	Test X^2^;p
Depression					
	No	n (%)	56 (46.3)	154 (63.6)	9.965;
	Yes	n (%)	65 (53.7)	88 (36.4)	0.002
Anxiety					
	No	n (%)	44(36.4)	145 (59.9)	17.931;
	Yes	n (%)	77 (63.6)	97 (40.1)	<0.001
Stress					
	No	n (%)	60 (49.6)	168 (69.4)	13.586;
	Yes	n (%)	61 (50.4)	74 (30.6)	<0.001

In the present study, there was no significant relationship of income level, cigarette and alcohol habits, and VDT use with DED. Considering general risk factors, there was a significant relationship of family history of DED (OR, 1.43; 95% CI, 0.84-2.41), chronic disease history (OR, 2.84; 95% CI, 1.66-4.87), OSDI score (OR, 1.07; 95% CI, 1.97–4.06), depression (OR, 2.06; 95% CI, 1.30-3.27), anxiety (OR, 2.66; 95% CI, 1.67-4.23), and stress (OR, 2.33; 95% CI, 1.48-3.67) with DED (Table-III). Adjusted OR and 95% Cl values for the risk factors identified in the univariate analysis are shown in Table-III.

**Table-III:** Adjusted OR and 95% Cl values for the risk factors identified in the univariate analysis.

		OR a (95% Cl)	p
Income level (reference: Good)			
	Fair	0.60 (0.25-1.47)	0.265
	Bad	1.24 (0.70-2.21)	0.474
Current Smoker (reference: No)			
	Yes	1.43 (0.84-2.41)	0.188
Current alcohol drinker (reference: No)			
	Yes	1.56 (0.31-7.92)	0.589
Family history of DED (reference: No)			
	Yes	3.63 (1.90-6.94)	<0.001
Chronic disease history (reference: No)			
	Yes	2.84 (1.66-4.87)	<0.001
VDT use (hours/day) (reference: No)			
	0-2	2.78 (0.31-24.7)	0.359
	2-4	6.21 (0.75-51.4)	0.091
	≥4	7.71 (0.92-64.7)	0.060
	OSDI score	1.07 (1.05-1.08)	<0.001
Depression (reference: No)			
	Yes	2.06 (1.30-3.27)	0.002
Anxiety (reference: No)			
	Yes	2.66 (1.67-4.23)	<0.001
Stress (reference: No)			
	Yes	2.33 (1.48-3.67)	<0.001

The dependent variable was the presence of defined DED and all of the associated factors identified in the univariate analyses (P < 0.2)

OR a: Odds ratio (adjusted by age, sex, and education), CI: Confidence interval.

## DISCUSSION

The present report is perhaps the first study regarding the prevalence of DED in Turkey; the prevalence of clinically diagnosed DED was 8.3% in 1458 outpatients. In hospital-based studies in some countries, the prevalence of DED varied between 7.99 and 29.25%.^7^-^11^ The variation between studies may be explained by the use of different diagnostic methods and age groups. In our study, we used the clinical diagnosis and studied individuals over 18 years of age, which could explain our finding of a lower prevalence of DED.

The present results confirmed the hypothesis that the risk of DED is increased for patients with depression, anxiety and/or stress (Table-III). A previous study showed that depression was associated with DED symptoms.^14^,^20^ Wang et al. have reported that patients with DED were more likely to have depression (OR 2.11).^20^ Several mechanisms can explain this relationship. The main explanations for this phenomenon are that dry eye symptoms may increase depression symptoms or antidepressant medications affect anticholinergic activity.^14^ Similar mechanisms are present for anxiety as well as depression. In accordance with the Wen et al. study, two case-control studies conducted by Li et al. showed that anxiety scores were correlated to dry eye symptoms.^15^,^21^ In addition, in the study by Wang et al., anxiety was not identified as a risk factor for DED.^20^ Stress that affects a significant part of a community can lead to changes in behaviour and physiology and can often lead to the development of psychological issues such as depression and anxiety.^22^ This study is the first study showing the relationship between stress and DED. This study showed that stress is an important risk factor for DED.

Various factors that contribute to the risk of dry eye have been proposed. Large epidemiological studies indicate that female sex and older age increase the risk for dry eye.^1^ Previous studies have demonstrated that the risk of DED is 1.56-1.85 times higher in females compared to males, and DED prevalence increases with age, most notably from the sixth decade.^4^,^6^,^10^,^23^ In the present study, the risk of DED was 3 times higher in individuals with a family history of DED. Family members share genes, lifestyles, and environments that together may influence their health. It is known that DED is associated with systemic hereditary diseases such as diabetes or autoimmune disorders.^20^,^23^ Furthermore, in our study, after the removal of the effects of age and sex on DED, chronic disease history was significantly associated with DED (OR 2.84). One reason for this association may be that the medications used for these chronic diseases may facilitate the occurrence of DED.

Recently, home use of computers and mobile devices has steadily increased. The widespread use of these devices in both young and older individuals is crucial to the increase in the incidence of DED in the general population. A study conducted by Uchino et al. reported that more than 4 hours of VDT use was associated with an increased risk of DED (OR 1.68).^24^ Li et al. have also reported similar results and determined that overexposure to VDTs is a major risk factor for DED among young individuals.^12^ In our study, there was no difference in VDT use between the case and control groups.

An assessment of the vision-targeted health-related quality of life, quantified by the OSDI, showed significantly better results in the control group. In accordance with the Li et al. and Labbe et al. studies, the present study shows that the severity of dry eye symptoms is an important variable for DED.^21^,^25^ This result is important because it shows that the OSDI can be used to diagnose DED without the effects of age and sex, and it can be used in population based studies without a clinical diagnosis.

The major limitations of this study are that it was a hospital-based study, and the scale for determining depression, anxiety and stress was self-reported.

In conclusion, to our knowledge, this is the first study reporting the prevalence of DED in Turkey. The prevalence of DED in our study using clinical diagnosis criteria is similar to that found in other studies of using the same criteria. Individuals with depression, anxiety and stress are more likely to experience DED. In addition to confirming some well-known risk factors, this study has found new associations between DED and a family history of DED and the presence of stress. Further population based studies evaluating the prevalence of DED in Turkey would be valuable.
